# Application of ImmunoScore Model for the Differentiation between Active Tuberculosis and Latent Tuberculosis Infection as Well as Monitoring Anti-tuberculosis Therapy

**DOI:** 10.3389/fcimb.2017.00457

**Published:** 2017-10-30

**Authors:** Yu Zhou, Juan Du, Hong-Yan Hou, Yan-Fang Lu, Jing Yu, Li-Yan Mao, Feng Wang, Zi-Yong Sun

**Affiliations:** ^1^Department of Laboratory Medicine, Tongji Hospital, Tongji Medical College, Huazhong University of Science and Technology, Wuhan, China; ^2^Wuhan Pulmonary Hospital, Wuhan Institute for Tuberculosis Control, Wuhan, China

**Keywords:** LTBI, ATB, ImmunoScore, diagnosis, therapy monitoring

## Abstract

Tuberculosis (TB) is a leading global public health problem. To achieve the end TB strategy, non-invasive markers for diagnosis and treatment monitoring of TB disease are urgently needed, especially in high-endemic countries such as China. Interferon-gamma release assays (IGRAs) and tuberculin skin test (TST), frequently used immunological methods for TB detection, are intrinsically unable to discriminate active tuberculosis (ATB) from latent tuberculosis infection (LTBI). Thus, the specificity of these methods in the diagnosis of ATB is dependent upon the local prevalence of LTBI. The pathogen-detecting methods such as acid-fast staining and culture, all have limitations in clinical application. ImmunoScore (IS) is a new promising prognostic tool which was commonly used in tumor. However, the importance of host immunity has also been demonstrated in TB pathogenesis, which implies the possibility of using IS model for ATB diagnosis and therapy monitoring. In the present study, we focused on the performance of IS model in the differentiation between ATB and LTBI and in treatment monitoring of TB disease. We have totally screened five immunological markers (four non-specific markers and one TB-specific marker) and successfully established IS model by using Lasso logistic regression analysis. As expected, the IS model can effectively distinguish ATB from LTBI (with a sensitivity of 95.7% and a specificity of 92.1%) and also has potential value in the treatment monitoring of TB disease.

## Introduction

Tuberculosis (TB) is a leading global public health problem with high morbidity and mortality in humans. As reported by the World Health Organization, there are almost 1.8 million deaths from TB in the year 2015. Meanwhile, of the estimated 10.4 million new active TB cases, India accounted for 23% and China accounted for 10% of the total (WHO, 2016). The development of new methods for diagnosis and treatment monitoring of TB is the key to control this disease.

Approximately one-third of the world's population is infected with Mycobacterium tuberculosis (Mtb). Most Mtb-infected individuals remain asymptomatic, with 5–10% developing active disease (Hoppe et al., 2016). Hence, the development of diagnostic tests which can distinguish active tuberculosis (ATB) from latent tuberculosis infection (LTBI) would be of great value. However, interferon-gamma release assays (IGRAs) and tuberculin skin test (TST), frequently used methods for TB detection, are unable to discriminate ATB from LTBI (Goletti et al., 2014). Other methods, such as microscopy of acid-fast staining (AFS) and culture for Mtb and nucleic acid detection technologies, all have limitations to meet the clinical requirements (Lewinsohn et al., 2016).

Many immunological markers are also evaluated for overcoming the problems (Dorhoi and Kaufmann, 2014; Hoppe et al., 2016). For instance, the expression of activation receptor HLA-DR (Fletcher et al., 2016) and immune inhibitory receptors PD-1 and Tim-3 (Blok et al., 2015; Wang et al., 2015; Jayaraman et al., 2016) on CD4+T cells or NK cells are significantly increased in ATB patients. Most existing studies focus on the role of immunological markers in the pathogenesis of TB disease, while evaluating host immune status as a way for ATB diagnosis is rarely concerned (Della Bella et al., 2008; Cantini et al., 2016; Harries et al., 2016; Petruccioli et al., 2016b). Two previous studies have shown that integrating multiple immune markers is better than using a single one for the discrimination of ATB and LTBI (Petruccioli et al., 2016a; Won et al., 2016). However, no useful models are established to analyze the effect of these immunological markers on TB diagnosis.

ImmunoScore (IS), mainly applied in the field of tumor, is now defined as a prognostic tool to use for quantification of *in situ* immune cell infiltrates (Galon et al., 2016; Mlecnik et al., 2016). The application of IS model to tumor prognosis further emphasizes the important role of host immunity in disease diagnosis and prognosis. Similarly, the host's immune status is significantly changed in the development of TB (O'Garra et al., 2013; Sia et al., 2015). Thus, we hypothesized that the IS model could also be used in the diagnosis and prognosis of TB disease.

To our knowledge, this is the first report of using TB-specific and non-specific markers to establish the IS model for identifying Mtb-infected individuals with different states. This study not only provides a more comprehensive insight into the host immune responses in the control of TB, but also offers an innovative method for ATB diagnosis and therapy monitoring.

## Materials and methods

### Study subjects and ethical approval

This study was carried out from 2016 to 2017 at Tongji Hospital, the largest hospital in central region of China. All the participants enrolled were classified into the following four categories: (1) healthy controls (HC); (2) LTBI; (3) ATB, including pulmonary tuberculosis (PTB) and extrapulmonary tuberculosis (EPTB); and (4) TR, the patients undergoing anti-TB treatment and showing good response.

Volunteers who had negative T-SPOT.TB results and without any pulmonary symptoms or active disease were recruited as HC. Individuals with positive T-SPOT.TB results but without clinical or radiographic evidence of ATB were diagnosed as LTBI. All ATB patients had positive T-SPOT.TB results, and they were categorized as follows: (1) confirmed ATB, smear positive or culture positive for Mtb or Mtb-specific PCR was positive; and (2) probable ATB, although Mtb was not identified in clinical samples, the clinical findings (including histopathologic, cytological, or biochemical indexes) were accordant with ATB and there was a positive response to anti-TB treatment. No ATB patients had started treatment at the time of enrolment. Classification of the enrolled participants was summarized in Supplementary Table 1. In the group of TR, blood samples of patients who had been treated with standard chemotherapy regimen (Hoppe et al., 2016) for one to 6 months were collected from Wuhan Pulmonary Hospital. Resolution of TB was assessed by clinical, radiological, and microbiological criteria (Supplementary Table 2). Patients with HIV or with solid organ transplantation or rheumatologic disease and receiving immunosuppressive treatment were excluded in the present study. The clinical and demographic characteristics of participants were shown in Table 1. This study was approved by the ethical committee of Tongji hospital, Tongji Medical College, Huazhong University of Science and Technology, Wuhan, China. All participants gave written consent to the study.

**Table 1:** Clinical and epidemiological characteristics of the study participants.

	**Training cohort (*****n*** = **127)**				**Testing cohort (*****n*** = **61)**		
	**HC (*n* = 20)**	**LTBI (*n* = 38)**	**ATB (*****n*** = **69)**		**LTBI (*n* = 27)**	**ATB (*****n*** = **34)**	
			**PTB (*n* = 32)**	**EPTB (*n* = 37)**		**PTB (*n* = 23)**	**EPTB (*n* = 11)**
Male sex	8 (40.0)	24 (63.2)	20 (62.5)	26 (70.3)	18 (66.7)	18 (78.3)	6 (54.5)
Age (mean±SD), years	35.8 ± 11.2	44.7 ± 9.9	46.1 ± 19.7	46.6 ± 14.7	44.1 ± 10.5	55.7 ± 17.6	41.8 ± 24.5
TB history	0 (0.0)	1 (2.6)	9 (28.1)	3 (8.1)	0 (0.0)	3 (13.0)	2 (18.2)
Confirmed ATB			10 (31.3)	7 (18.9)		11 (47.8)	2 (18.2)
Smear			5 (15.6)	1 (2.7)		3 (13.0)	1 (9.1)
Culture			9 (28.1)	6 (16.2)		8 (34.8)	1 (9.1)
PCR			1 (3.1)	1 (2.7)		6 (26.1)	1 (9.1)
Probable ATB			22 (68.8)	30 (81.1)		12 (52.2)	9 (81.8)
**INFECTION SITE**							
Lung			32 (100.0)			23 (100.0)	
Pleura				15 (40.5)			5 (45.5)
Lymph				6 (16.2)			1 (9.1)
Bone				6 (16.2)			1 (9.1)
Urinary				3 (8.1)			0 (0.0)
Skin				3 (8.1)			1 (9.1)
Intestinal				2 (5.4)			2 (18.2)
Others				2 (5.4)			1 (9.1)

*Data are presented as numbers (%) unless otherwise indicated. HC, healthy controls; LTBI, latent tuberculosis infection; ATB, active tuberculosis; PTB, pulmonary tuberculosis; EPTB, extrapulmonary tuberculosis*.

### PBMCs isolation and stimulation

Samples of peripheral blood were collected from all participants. Peripheral blood mononuclear cells (PBMCs) were separated by Ficoll-Hypaque density gradient centrifugation. To determine the functional potential of CD4+ T cells, CD8+ T cells, NK cells, dendritic cells (DC) and monocytes (MON), phorbol-12-myristate-13-acetate (PMA) (50 ng/ml; Sigma) and ionomycin (1 μg/ml, Sigma), phytohaemagglutinin (PHA) (10 μg/ml, Oxford Immunotec), lipopolysaccharides (LPS) (10 μg/ml, Sigma) were used to stimulate PBMCs, respectively.

### Fluorescence labeling and flow cytometric analysis

After stimulation, PBMCs were collected and the following surface antibodies were added to the cell suspensions in different tubes: anti-CD3-PerCP-Cy5.5 (OKT3, BioLegend), anti-CD4-APC-Cy7 (RPA-T4, BioLegend), anti-CD8-FITC (HIT8a, BioLegend), anti-CD8-PE (OKT4, BioLegend), anti-CD14-APC-Cy7 (HCD14, BioLegend), anti-CD19-APC-Cy7 (HIB19, BioLegend), anti-CD56-PE-Cy7 (MEM-188, BioLegend), anti-CD25-PE (M-A251, BioLegend), anti-HLA-DR-APC (H4A3, BioLegend), anti-HLA-DR-FITC (L243, BioLegend), anti-CD107a-FITC (OKT4, BioLegend), Anti-PD-1-FITC (NAT105, BioLegend), anti-Tim-3-APC (F38-2E2, BioLegend) and anti-CD45-V450 (HI30, BD Bioscience). Isotype controls (ISO) with irrelevant specificities were included as negative controls. The different cell subsets were gated as the following criteria: (1) CD4+ and CD8+ T cells were characterized as CD3+CD4+ and CD3+CD8+ subsets; (2) NK cells were gated as CD3^−^CD56+ subsets; (3) MON were gated as CD45+CD14+HLA-DR+ subsets; and (4) DC were characterized by the expression of HLA-DR and negative expression of CD3, CD19 and CD56 (Supplementary Figure 1). All the cell suspensions were incubated for 20 min in the dark at room temperature. For intracellular staining, the cells were collected after surface staining. After washings, the cells were fixed and permeabilized and stained with anti-IFN-γ-PE (B27, BD Bioscience), anti-IFN-γ-APC (B27, BD Bioscience), anti-TNF-α-PE (MAb11, BD Bioscience), anti-TNF-α-APC (MAb11, BD Bioscience) and anti-IL-2-PE (MQ1-17H12, BD Bioscience) for 20 min in the dark. After washings, the pellets were resuspended in 200 μl PBS buffer and analyzed with FACSCalibur and FACSCanto flow cytometers (Becton Dickinson). Data analysis was performed through FlowJo version 7.6.1 software (TreeStar).

#### T-SPOT.TB assay and TBAg/PHA calculation

The T-SPOT.TB assay was performed according to the manufacturer's instructions (Oxford Immunotec, Oxford, England). The TBAg/PHA ratio of T-SPOT.TB assay used as TB-specific marker in the present study was calculated as previously described (Wang et al., 2016; Bosco et al., 2017). Briefly, the calculated ratios of (1) early secreted antigenic target 6 (ESAT-6) spot-forming cells (sfc) to PHA sfc, and (2) culture filtrate protein 10 (CFP-10) sfc to PHA sfc were compared and the larger of these two values was defined as the TBAg/PHA ratio of one individual.

### Statistical analysis

The statistical differences between different groups were analyzed using unpaired Student's *t*-test. Receiver-operating-characteristic (ROC) curves were used to determine the cut-off values of the different factors for distinguishing ATB from LTBI. Area under the curve (AUC) and optimal combination of sensitivity and specificity (highest sum of sensitivity and specificity) were reported, as well as the 95% confidence intervals (CI) of the AUC. Statistical significance was determined as *P* < 0.05.

The least absolute shrinkage and selection operator method (Lasso) was employed to establish logistic regression model. Combined with professional knowledge, the Lasso logistic regression was used to select the most useful indicators out of all the tested immunological features and constructed multi-immune feature-based classifiers for ATB diagnosis. The “glmnet” package performed through R software (version 3.3.3) was used to conduct the Lasso logistic regression model analysis. Graphpad Prism 5.01 (GraphPad Software, CA, USA) was used for plotting the data. All other statistical tests were performed with SPSS software (version 19.0).

## Results

### Participant characteristics

The main characteristics of participants were shown in Table 1. There were 20 HC, 38 LTBI and 69 ATB patients enrolled for immune function evaluation and IS model establishment. There was no significant difference in sex and age distribution between ATB and LTBI, while the average age and male' rate of ATB group were higher than HC group (*p* < 0.05). In the training cohort, 31.3% of PTB and 18.9% of EPTB had confirmed diagnosis of ATB. Tuberculous pleuritis (40.5%) was the major form of EPTB. For validation of the IS model, another 61 participants were enrolled as testing group. In this group, there was no significant difference in sex and age between ATB and LTBI, and 47.8% of PTB, 18.2% of EPTB had confirmed diagnosis of ATB.

#### Impaired cell function in ATB patients

**Figure 1:**
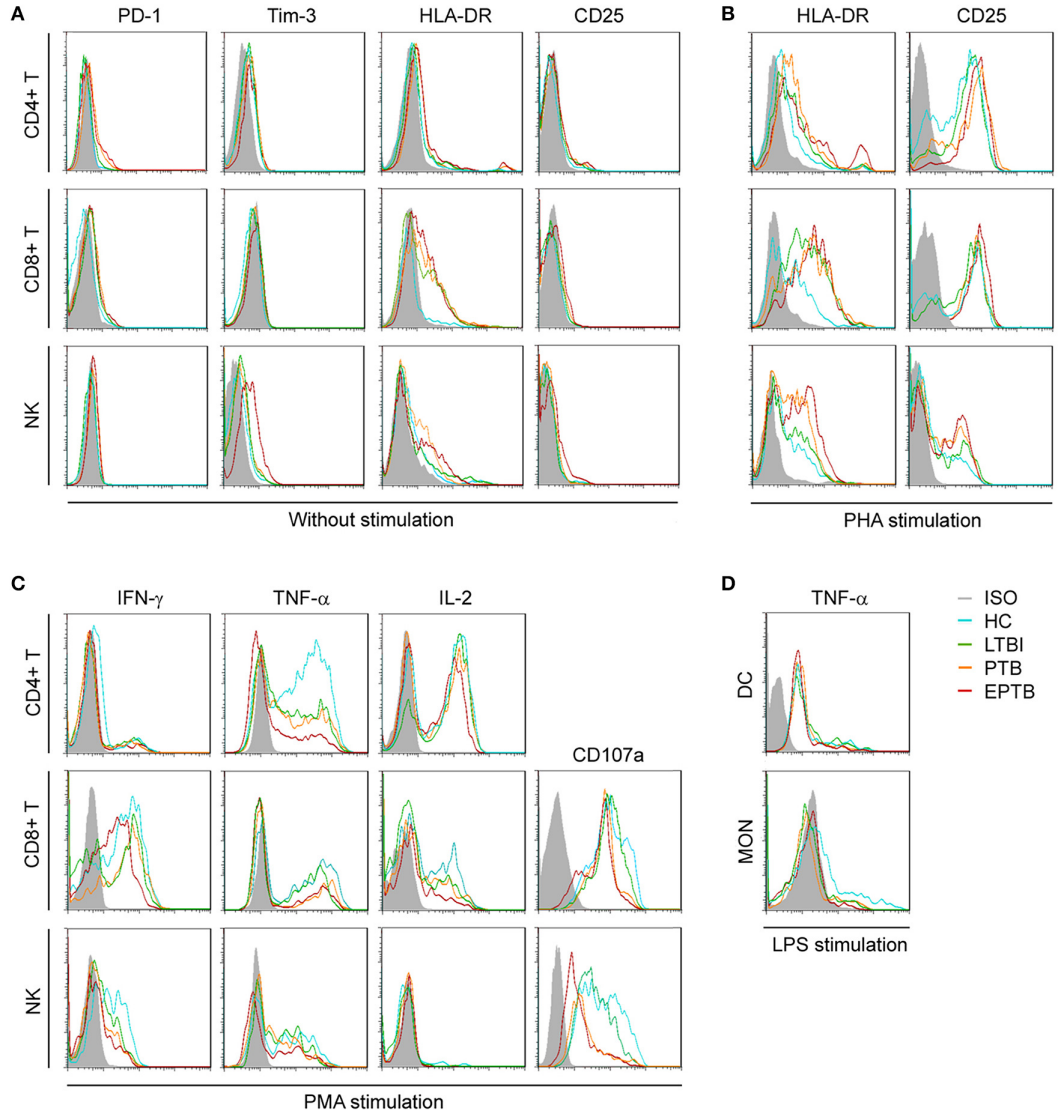
Representative FACS histograms showing the expression of TB non-specific immunological markers. Inhibitory receptors (PD-1, Tim-3) **(A)**, activation receptors (HLA-DR, CD25) **(A,B)**, cytotoxicity marker (CD107a) **(C)**, and intracellular cytokines (IFN-γ, TNF-α, IL-2) on non-stimulated or PHA-, PMA-, or LPS-stimulated CD4+ T cells, CD8+ T cells, NK cells, DC, and MON **(C,D)** in HC, LTBI, PTB, and EPTB patients were evaluated by flow cytometer. ISO, isotype controls; HC, healthy controls; LTBI, latent tuberculosis infection; ATB, active tuberculosis; PTB, pulmonary tuberculosis; EPTB, extrapulmonary tuberculosis.

**Figure 2:**
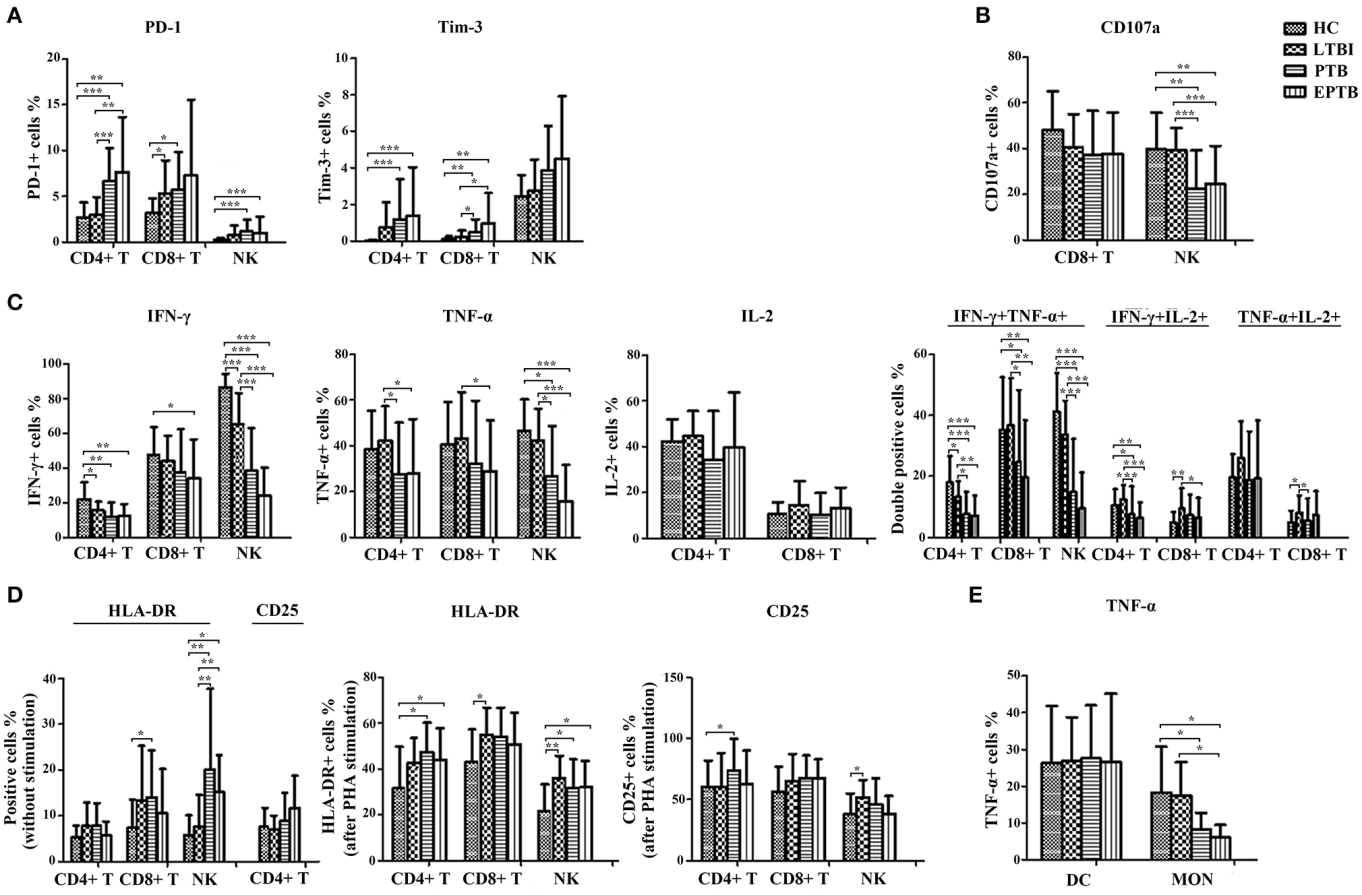
The statistical results of the expression of non-specific immunological markers. Samples of peripheral blood were collected from HC (*n* = 20), LTBI (*n* = 38), PTB (*n* = 32), and EPTB (*n* = 37). The percentages of indicators on different immune cells were analyzed by flow cytometry. Results are expressed as the mean ± SD. **(A)** The percentages of PD-1+ and Tim-3+ CD4+ T, CD8+ T and NK cells in different groups were shown (without stimulation). **(B)** The percentages of CD107a+ CD8+ T and NK cells in different groups were shown (PMA stimulation). **(C)** The percentages of single IFN-γ+, TNF-α+, IL-2+, or IFN-γ+/TNF-α+, IFN-γ+/IL-2+, TNF-α+/IL-2+ CD4+ T, CD8+ T and NK cells in different groups were shown (PMA stimulation). **(D)** The percentages of HLA-DR+ and CD25+ CD4+ T, CD8+ T and NK cells in different groups were shown (without stimulation or PHA stimulation). **(E)** The percentages of TNF-α+ DC and MON in different groups were shown (LPS stimulation). ^*^p < 0.05, ^**^p < 0.01, ^***^p < 0.001. HC, healthy controls; LTBI, latent tuberculosis infection; ATB, active tuberculosis; PTB, pulmonary tuberculosis; EPTB, extrapulmonary tuberculosis.

To evaluate the immune status of Mtb-infected individuals, the main non-specific immunological markers were determined in different groups. The expression of typical non-specific immunological markers on different cell subsets was shown in Figure 1. Our results showed that the expression of inhibitory receptors PD-1 and Tim-3 was increased in PTB patients. The levels of PD-1 expression on CD4+ T cells and Tim-3 expression on CD8+ T cells in PTB patients were significantly higher than those in LTBI individuals or HC (Figure 2A). Similarly, the expression of activation receptor HLA-DR on NK cells in PTB patients was significantly increased compared with LTBI individuals or HC (Figure 2D). In contrast, the cytolytic activity and cytokine secretion potential of lymphocytes in PTB patients was decreased. The percentages of IFN-γ+ and IFN-γ+TNF-α+ CD4+T cells, and CD107a+, IFN-γ+, TNF-α+ and IFN-γ+TNF-α+ NK cells in PTB patients were significantly decreased compared with LTBI individuals or HC (Figures 2B,C). The TNF-α secretion potential of MON was also significantly decreased in PTB patients compared with HC (Figure 2E). Furthermore, compared with PTB patients, although EPTB patients had a decreased trend of cell function, no statistical difference was found in these immunological markers. These data suggest that the impaired cell function may lead to the development of TB.

### Potential value of the TBAg/PHA ratio for differentiation of different TB states

**Figure 3:**
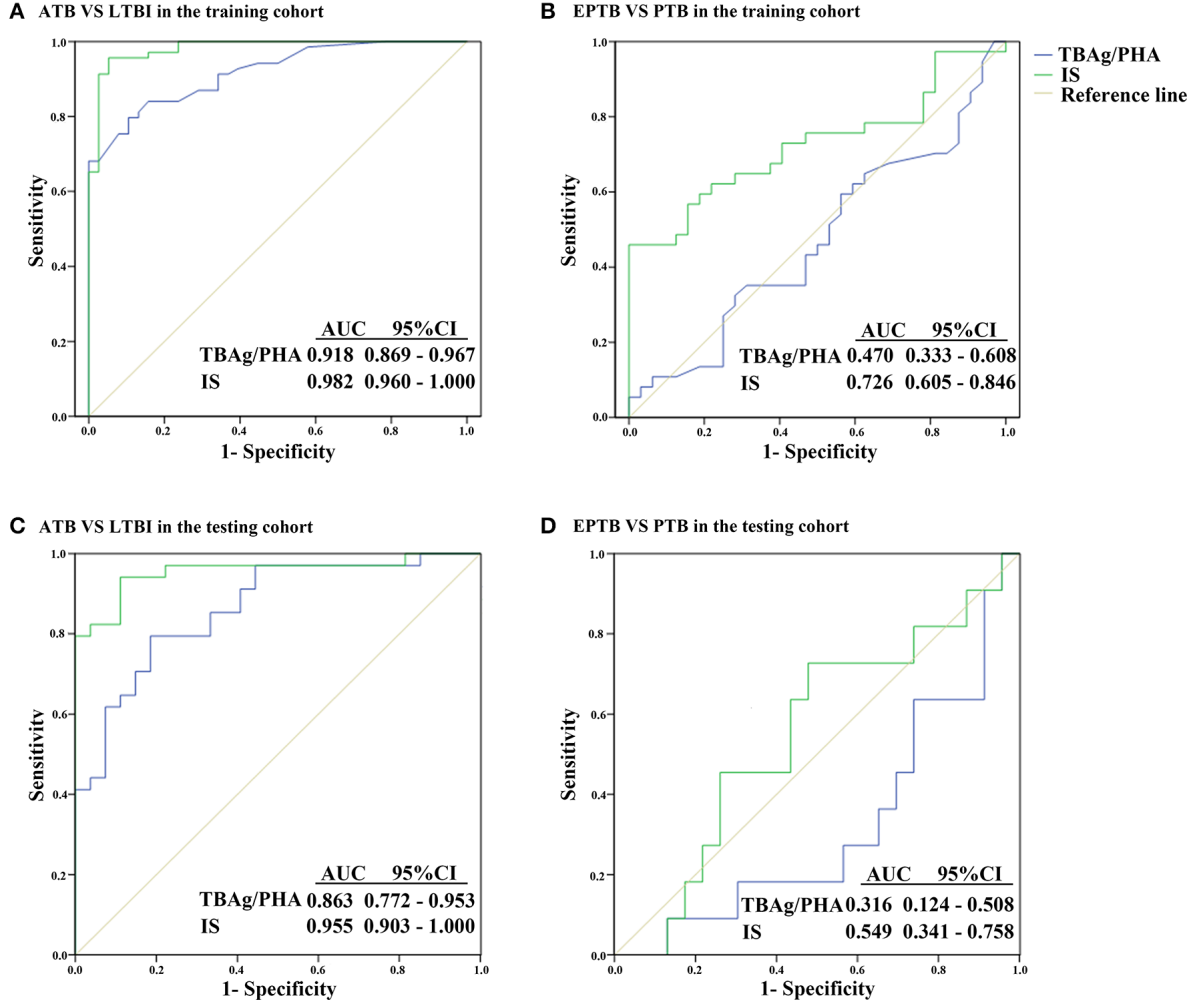
ROC analysis of the TBAg/PHA ratio and IS model for distinguishing different TB disease states. **(A)** ROC analysis of the TBAg/PHA ratio and IS for ATB and LTBI discrimination in the training cohort. **(B)** ROC analysis of the TBAg/PHA ratio and IS for EPTB and PTB discrimination in the training cohort. **(C)** ROC analysis of the TBAg/PHA ratio and IS for ATB and LTBI discrimination in the testing cohort. **(D)** ROC analysis of the TBAg/PHA ratio and IS for EPTB and PTB discrimination in the testing cohort. ATB, active tuberculosis; LTBI, latent tuberculosis infection; EPTB, extrapulmonary tuberculosis; PTB, pulmonary tuberculosis; AUC, area under the curve.

As the level of TB-specific IFN-γ and TNF-α secretion (the most relevant cytokines for TB diagnosis at present) was quite low by flow cytometric detection (Supplementary Figure 2), TB-specific immunological marker was based on IFN-γ ELISPOT assay (T-SPOT.TB). Previous studies have shown that the TBAg/PHA ratio of T-SPOT.TB has potential in distinguishing ATB from LTBI (Wang et al., 2016). Thus, the TBAg/PHA ratio was defined as TB-specific marker and was evaluated in the present study. The AUC of the ROC curve for the TBAg/PHA ratio was 0.918, with a sensitivity of 79.7% and a specificity of 89.5% when a threshold value of 0.245 was used to discriminate between ATB and LTBI (Figure 3A, Table 2). The value of the TBAg/PHA ratio in distinguishing EPTB from PTB was very limited, with a sensitivity of 35.1% and a specificity of 67.8% if using the cut-off value of 0.985 (Figure 3B, Table 2).

**Table 2:** Performance of the TBAg/PHA ratio and IS for discriminating between ATB and LTBI or between EPTB and PTB.

	**Cut-off value**	**Sensitivity**	**Specificity**
**TRAINING COHORT**			
**ATB vs. LTBI**			
TBAg/PHA ratio	0.245	79.7%	89.5%
IS	0.175	95.7%	92.1%
**EPTB vs. PTB**			
TBAg/PHA ratio	0.985	35.1%	68.7%
IS	0.123	56.8%	84.4%
**TESTING COHORT**			
**ATB vs. LTBI**			
TBAg/PHA ratio	0.129	79.4%	81.5%
IS	0.180	94.1%	88.9%
**EPTB vs. PTB**			
TBAg/PHA ratio	0.523	18.2%	69.6%
IS	1.798	72.7%	52.2%

*ATB, active tuberculosis; LTBI, latent tuberculosis infection; PTB, pulmonary tuberculosis; EPTB, extrapulmonary tuberculosis*.

### Is model for discriminating ATB and LTBI

Although the TBAg/PHA ratio showed potential in ATB and LTBI discrimination, its sensitivity was relatively low as a diagnostic method. Lasso logistic regression analysis was used to determine whether the combination of TB-specific and non-specific immunological markers could improve the diagnostic effect. The IS model for ATB identification generated by Lasso logistic regression analysis using 10-fold cross-validation eventually selected five immunological indicators: IS = (0.597 ^*^ TBAg/PHA ratio) + (0.042 ^*^ PD-1+CD4+ T %) + (0.028 ^*^ HLA-DR+ NK %) − (0.052 ^*^ CD107a+ NK %) − (0.029^*^ IFN-γ+ NK %) + 2.446 (Table 3). The ROC analysis showed the performance of IS in distinguishing ATB from LTBI was improved. The AUC of the ROC curve was 0.982. Using 0.175 as the cut-off value, the sensitivity and specificity were 95.7 and 92.1% (Figure 3A, Table 2). These data suggest that the IS model has a good performance in distinguishing ATB from LTBI.

**Table 3:** IS model for discrimination between ATB and LTBI.

**Indicators**	**Coefficients**
Intercept	2.446
TBAg/PHA ratio	0.597
PD-1+ CD4+ T %	0.042
HLA-DR+ NK %	0.028
CD107a+ NK %	−0.052
IFN-γ+ NK %	−0.029

*ATB, active tuberculosis; LTBI, latent tuberculosis infection*.

### Using IS model to distinguish EPTB from PTB

Given that the cell function in EPTB patients had a decreased trend compared with PTB patients, the potential value of the IS model in discrimination of EPTB and PTB was also evaluated. Employed with Lasso logistic regression, the IS model was slightly different in this part, while only two indicators were included: IS = (0.049 ^*^ PD-1+CD4+ T %) − (0.005 ^*^ IFN-γ+ NK %) − 0.263 (Table 4). Using ROC analysis of IS model in distinguishing EPTB from PTB, the AUC of the ROC curve was 0.726, with a sensitivity of 56.8% and a specificity of 84.4% at the cut-off value of 0.123 (Figure 3B, Table 2). These data suggest the value of IS for differentiation of EPTB and PTB is very limited.

**Table 4:** IS model for discrimination between EPTB and PTB.

**Indicators**	**Coefficients**
Intercept	−0.263
TBAg/PHA ratio	—
PD-1+CD4+ T %	0.049
HLA-DR+ NK %	—
CD107a+ NK %	—
IFN-γ+ NK %	−0.005

*PTB, pulmonary tuberculosis; EPTB, extrapulmonary tuberculosis*.

### Validation of IS model for ATB diagnosis

To validate the performance of IS model in the diagnosis of ATB, the testing cohort was composed of 27 LTBI individuals and 34 ATB patients. The sensitivity and specificity of the TBAg/PHA ratio in distinguishing ATB from LTBI were 79.4 and 81.5%, respectively. When using IS model, the sensitivity and specificity in distinguishing these two conditions were 94.1 and 88.9%, respectively (Figure 3C, Table 2). These data suggest that the performance of IS model in the testing cohort is similar with the training cohort.

The value of IS model in EPTB and PTB discrimination was also evaluated in the testing cohort. We found the performance of IS model in the testing cohort was worse than that in the training cohort. If using cut-off value of 1.798, the sensitivity and specificity of IS model in distinguishing EPTB from PTB were 72.7 and 52.2%, respectively (Figure 3D, Table 2).

### Application of IS model in prognosis evaluation

**Figure 4:**
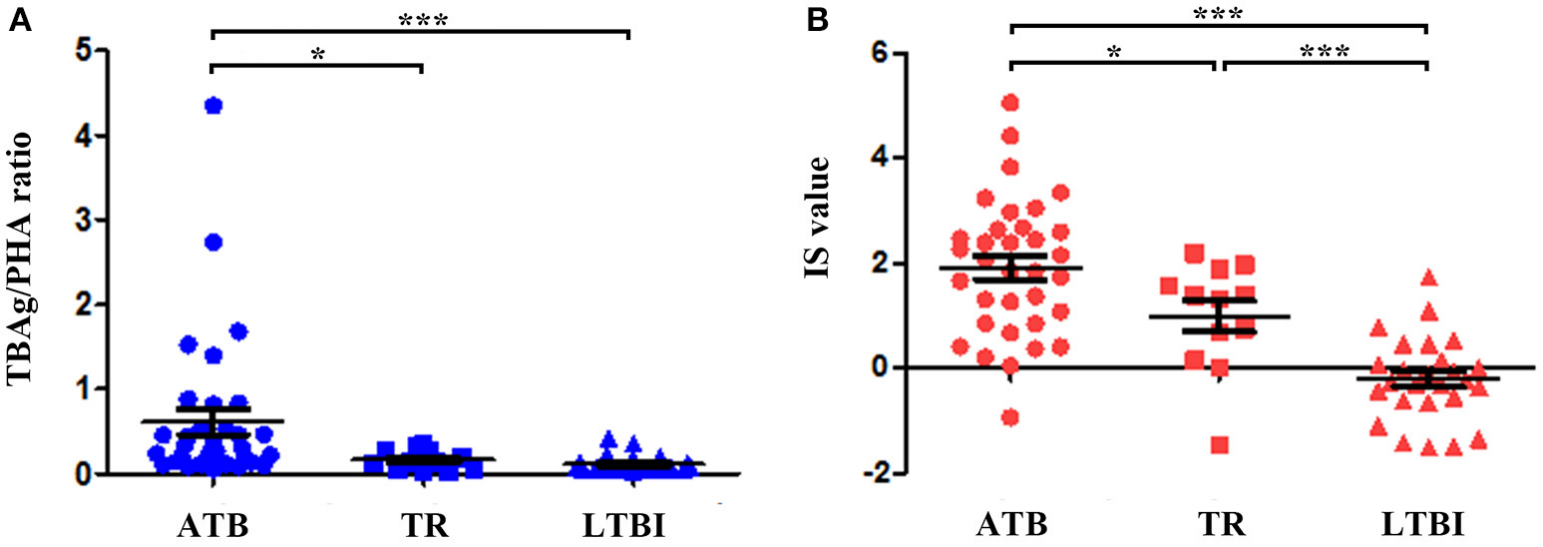
The value of the TBAg/PHA ratio and IS model for monitoring anti-TB therapy. **(A)** Dot plots showing the TBAg/PHA ratio in different groups (ATB, TR, LTBI). **(B)** Dot plots showing the value of IS model in different groups (ATB, TR, LTBI). ^*^p < 0.05, ^***^p < 0.001. Error bars indicate mean ± SEM. ATB, active tuberculosis; TR, the patients undergoing anti-TB treatment; LTBI, latent tuberculosis infection.

The quantitative change of the IS value during anti-TB treatment might reflect therapeutic efficacy. There were 12 TR enrolled for IS evaluation. Our results showed that after one to 6 months of anti-TB treatment, both the TBAg/PHA ratio and IS value were significantly decreased in TR group compared with ATB group. However, although the IS value was gradually reduced during anti-TB treatment, it was still higher than that in LTBI group. These data show that the IS model can reflect the change of immune status during therapy, which suggests the IS model has the potential in monitoring anti-TB treatment (Figure 4).

## Discussion

Finding new Methods which could predict the risk of developing ATB and the response to anti-TB treatment is major goal for TB control (Goletti et al., 2016). Although reports on new candidate markers are numerous, there is rarely one marker which is suitable for clinical use. Meanwhile, only scant information exists regarding the combination of different immunological markers in the diagnosis of TB disease. In the present study, by the combination of non-specific immunological markers and TB-specific marker (TBAg/PHA ratio), we successfully established the IS model that not only shows good performance in distinguishing ATB from LTBI but also has potential in monitoring anti-TB treatment.

IS, which appears to be superior to the tumor-node-metastasis classification (Mlecnik et al., 2016; Petrizzo and Buonaguro, 2016), is now defined as a prognostic tool in many different types of tumors. The natural history of cancer involving interactions between tumor and the host immune system makes IS a promising method for monitoring tumor progression. Similarly, the interactions between Mtb and host immunity are also critical for TB development. The Mtb-infected individuals with different immune status commonly lead to very different outcomes (O'Garra et al., 2013; Serrano et al., 2015), which supports the feasibility of using IS model for TB diagnosis and prognosis.

In the present study, evaluation of TB non-specific immunological markers showed that the cell function of ATB patients was impaired compared with that of LTBI or HC. In consistent with our results, Qiu et al. reported that the frequency of TB non-specific multifunctional CD4+ T cells was decreased in ATB patients (Hao et al., 2012). Besides, PD-1 is a critical regulatory molecule, which mediates T cell exhaustion in many chronic infections (Parida and Kaufmann, 2010; Day et al., 2011; Su et al., 2014). Our results showed that the expression of PD-1 was significantly increased in ATB patients, which is in accordance with other studies (Jurado et al., 2012; Hassan et al., 2015). Lysosome-associated glycoprotein CD107a serves as a marker of degranulation of CD8+ T cells and NK cells (Aktas et al., 2009; Shabrish et al., 2016). Kumar et al. (2015) and our study Wang et al. (2015) found that CD107a expression on CD8+ T cells and NK cells is significantly diminished in ATB patients, which indicates the impairment of these cells during TB progression. Furthermore, CD25 and HLA-DR are typical immunological markers that reflect T cell activation in response to microbial infection or vaccination (Sava and Toldi, 2016; Bajnok and Ivanova, 2017). Our results showed that the basal expression level of HLA-DR on NK cells was high in ATB patients, which suggests that the activated immune response is sustained in TB disease and might further explain why the immune cells are exhausted in ATB patients (Khan et al., 2017).

The classical pro-inflammatory cytokines, particularly of IFN-γ, IL-2, and TNF-α, serve as key components in the control of and protection against Mtb infection (O'Garra et al., 2013; Serrano et al., 2015). However, after stimulation, we observed that the secretion of IFN-γ and TNF-α by NK cells was remarkably decreased in ATB patients. In general, NK cells are not only the key effectors in the innate immune response to various infections (Bapat et al., 2015), but are also the effective regulators in innate and adaptive immune responses (Deng et al., 2014). In accordance with previous studies (Kim et al., 2015; Pan et al., 2015), our results indicate that the impaired cytokine production by NK cells may be an important factor in TB development. Moreover, multifunctional T cells, which are defined by their ability to co-express two or more cytokines, show an improved discrimination between ATB and LTBI (Caccamo et al., 2010). Many reports suggest that multifunctional TB-specific CD4+ T cells play an important role in protective immunity against Mtb infection (Day et al., 2006; Prezzemolo et al., 2014; Panteleev et al., 2017). Our results also showed that an obvious decrease of IFN-γ/TNF-α or IFN-γ/IL-2 double positive NK cells or CD4+ T cells was observed in ATB patients, which emphasizes the important role of multifunctional T cells and NK cells in the control of TB. Overall, the functional potential of lymphocytes is impaired in TB disease, which may lead to the development of TB.

T-SPOT.TB assay, which depends on the detection of IFN-γ in response to TB antigens, is widely used for immunological diagnosis of Mtb infection. However, a great limitation of this assay is its inability to distinguish ATB from LTBI (Borgstrom et al., 2012; Rajaram et al., 2014). To overcome this problem, our previous studies have proposed that a further calculation of the TBAg/PHA ratio can eliminate the impact of individual immune variations on T-SPOT.TB assay, which is better than directly using T-SPOT.TB results in distinguishing these two conditions (Wang et al., 2016). In addition, because of low level of IFN-γ secretion after TB-specific antigen stimulation, it is difficult to detect this cytokine by flow cytometry. Thus, the TBAg/PHA ratio was defined as TB-specific immunological marker in the present study. Actually, some researches have concerned the combination of T-SPOT.TB and immunological markers in the diagnosis of TB. Yang et al. once reported that in combination with T-SPOT.TB assay, CD161-expressing T cells have potential in distinguishing ATB from LTBI. However, this method achieves only a sensitivity of 76.6% and a specificity of 90.5% (Yang et al., 2015). Meanwhile, another study suggested that Mtb-specific IFN-γ and IL-2 ELISPOT assays may be helpful in discriminating children with active or latent tuberculosis (Cardona et al., 2012). However, it is not clear whether this conclusion is applicable to adults, and the authors did not determine the combination effect of these markers on TB diagnosis.

Given that many immunological markers are changed in the development of TB, how to screen the most effective markers for TB diagnosis is important. By using the Lasso logistic regression, we have successfully screened five markers and further established the IS model. Immunological markers selected in the IS model are TB-specific marker (TBAg/PHA ratio), inhibitory receptor (PD-1+CD4+ T %), activation receptor (HLA-DR+ NK %), cytotoxicity marker (CD107a+ NK %), and cytokine secretion capacity (IFN-γ+ NK %). These included markers are closely related to TB progression as mentioned above. Our established IS model shows good performance in distinguishing ATB from LTBI, with a sensitivity of 95.7% and a specificity of 92.1% at the IS value of 0.175. Although many researchers focus on the immunological markers in the pathogenesis of TB, no one uses these markers to establish IS model for TB diagnosis. To our knowledge, this is the first report of the use of IS model in the diagnosis of TB disease, while this model has been considered as a useful tool for evaluation of tumor prognosis (Galon et al., 2016).

Currently, evaluation of the effect of anti-TB treatment is difficult in clinical practice due to the lack of reliable tools. Although several cytokine markers are found to have potential application in monitoring anti-TB treatment, no single or combination of cytokines demonstrates a robust correlation with treatment outcome (Hur et al., 2015; Chen et al., 2016). In the present study, we observed that both the TBAg/PHA ratio and IS value exhibited a significant decrease in TR group compared with ATB group, which suggests the potential use of IS model in the treatment monitoring of TB. Furthermore, the TBAg/PHA ratio showed more significant change than IS value during anti-TB treatment, which could be due to more sensitivity of TB-specific immune response to the positive treatment outcome. Although further validation studies are required, our primary results suggest the probable application of IS model for monitoring anti-TB treatment.

Although the results are promising, there are some limitations should be mentioned. First of all, this is a single center study with a small sample size. This could generate bias in the model establishment. In order to verify the diagnostic value of the model, future validation studies in various ethnic groups and multi-centers with large numbers of patients are necessary. Secondly, dynamic tracking of immune function in certain patients, especially in LTBI who are developing active disease, or in those undergoing anti-TB treatment, is a better way to evaluate the performance of IS model and will be more helpful to guide individualized treatment. Finally, although multicolor flow cytometry is able to test all the selected immune markers conveniently, the isolation of PBMCs is still time-consuming. Therefore, in order to facilitate clinical practice, we will try to evaluate the use of whole blood instead of PBMCs as well.

## Conclusions

This study demonstrates that the normal host immunity is involved in controlling Mtb infection and that the impairment of cell function is the key factor to cause TB development. By combination of the immunological markers, the established IS model has the potential in distinguishing ATB from LTBI and monitoring anti-TB treatment. If results from this study are found to be replicable in other settings, this IS model might provide a useful tool for the diagnosis and prognosis of TB disease.

## Author contributions

FW and ZS designed the study. HH, YL, and JY were responsible for recruitment, interview of the patients, samples collection and transport to the laboratory. YZ was in charge of laboratory procedures. LM did the statistical analysis. YZ, FW, and ZS wrote the manuscript. All authors read and approved the final manuscript.

### Conflict of interest statement

The authors declare that the research was conducted in the absence of any commercial or financial relationships that could be construed as a potential conflict of interest.

## Supplementary material

Supplementary Figure 1 Flow cytometry gating strategy for CD4+ T cells, CD8+ T cells, NK cells, DC, and MON. Leukocytes were gated according to CD45 expression and side scatter (SSC). **(A)** CD4+ and CD8+ T cells were gated as CD3+CD4+ and CD3+CD8+ cells; **(B)** NK cells were gated as CD3-CD56+ cells; **(C)** DC were gated as CD3-CD19-CD56-HLA-DR+ cells; **(D)** MON were gated as CD45+CD14+HLA-DR+ cells.

Supplementary Figure 2 The secretion of TB-specific cytokines by lymphocytes analyzed with flow cytometry. The PBMCs isolated from ATB patients were stimulated with TB-specific antigens (ESAT-6 and CFP-10) for 24 h. After stimulation, the cells were collected for analysis of intracellular IFN-γ and TNF-α in CD4+ T, CD8+ T and NK cells.
