# Supplementary figures and images for: Application of ImmunoScore Model for the Differentiation between Active Tuberculosis and Latent Tuberculosis Infection as Well as Monitoring Anti-tuberculosis Therapy
Source: Front Cell Infect Microbiol. 2017 Oct 30;7:457. doi: 10.3389/fcimb.2017.00457 (PMC5670161; doi:10.3389/fcimb.2017.00457)

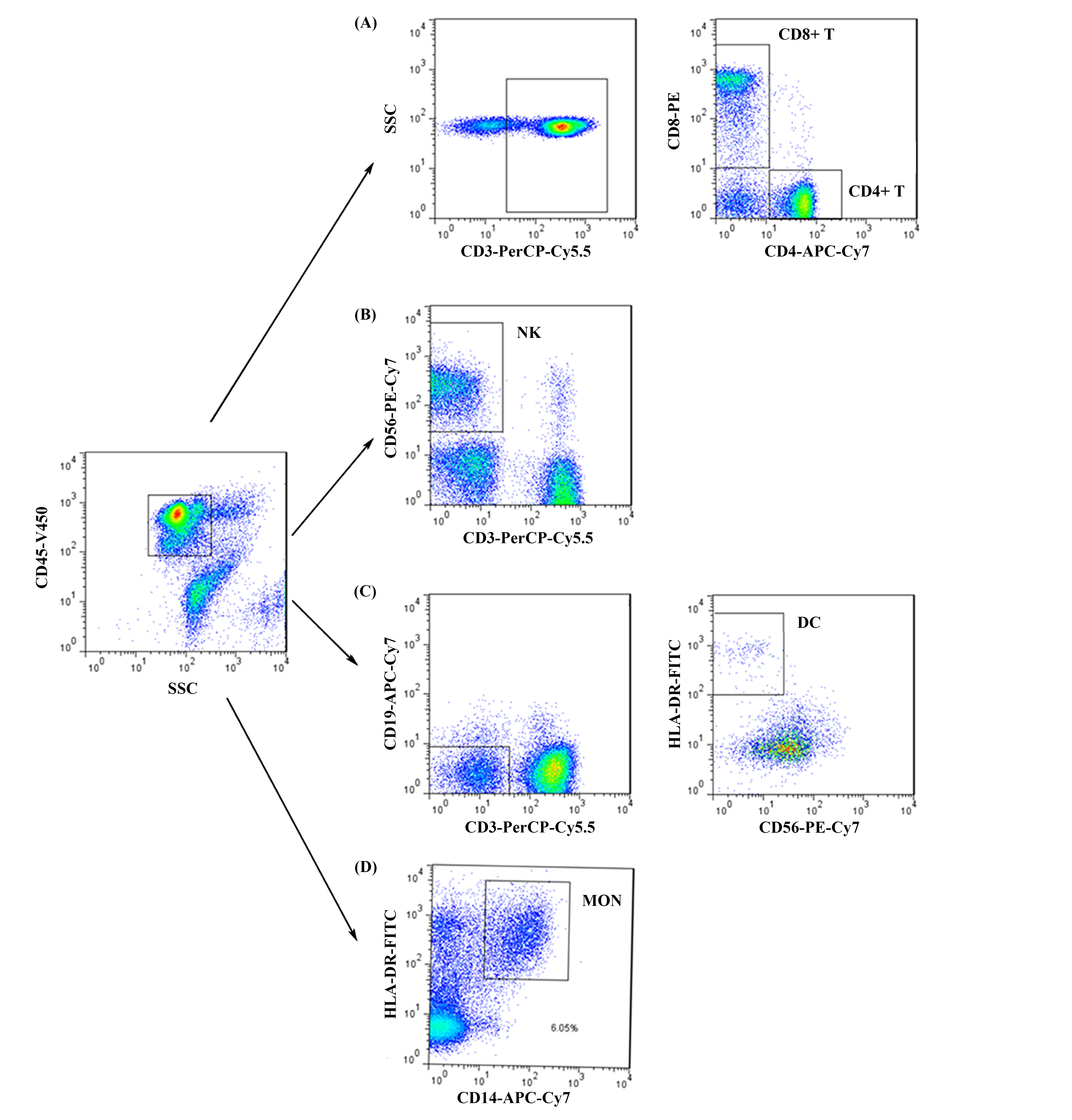

Supplement: Supplementary Figure 1 — Flow cytometry gating strategy for CD4+ T cells, CD8+ T cells, NK cells, DC, and MON. Leukocytes were gated according to CD45 expression and side scatter (SSC). (A) CD4+ and CD8+ T cells were gated as CD3+CD4+ and CD3+CD8+ cells; (B) NK cells were gated as CD3-CD56+ cells; (C) DC were gated as CD3-CD19-CD56-HLA-DR+ cells; (D) MON were gated as CD45+CD14+HLA-DR+ cells. [file Image1.TIF]

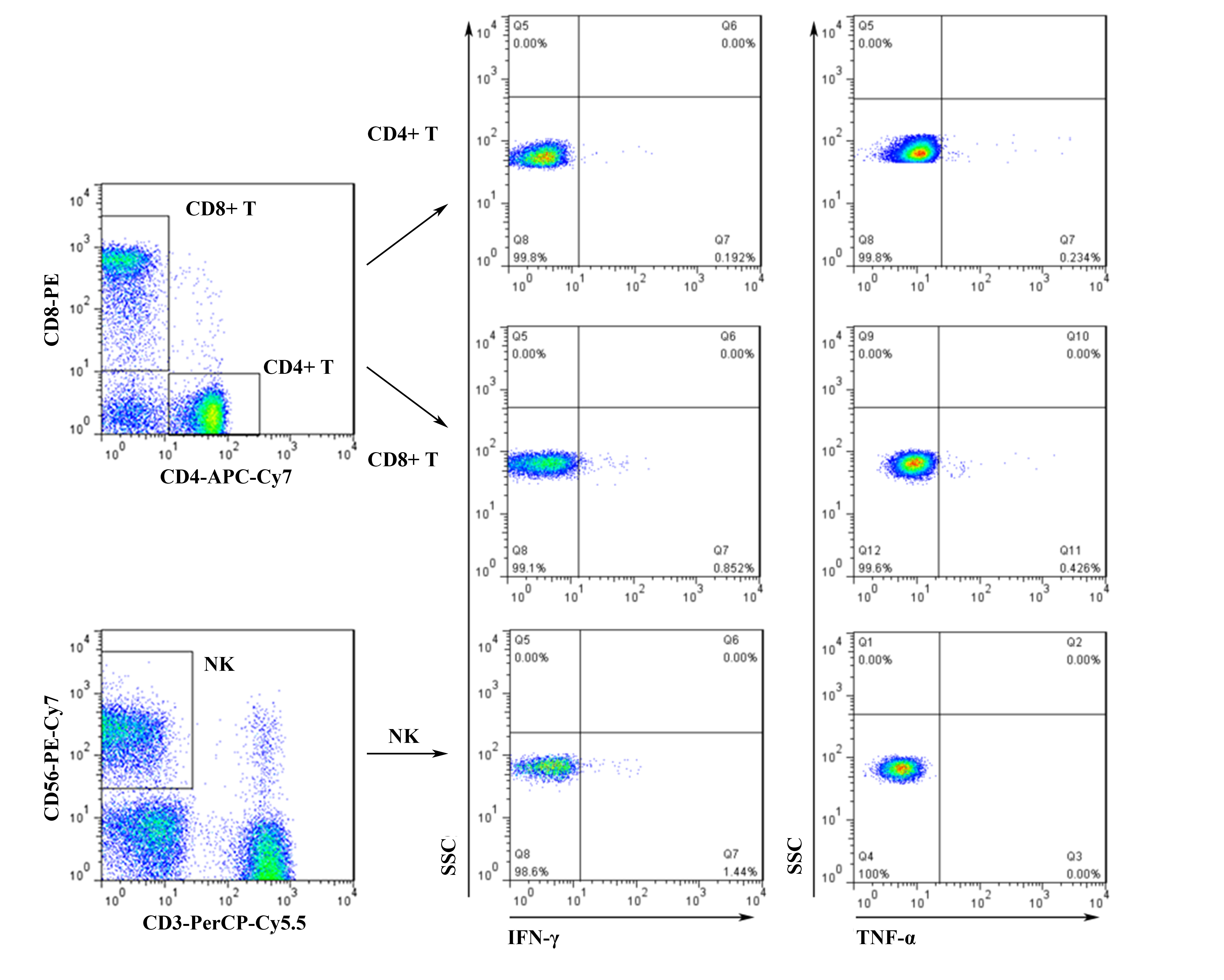

Supplement: Supplementary Figure 2 — The secretion of TB-specific cytokines by lymphocytes analyzed with flow cytometry. The PBMCs isolated from ATB patients were stimulated with TB-specific antigens (ESAT-6 and CFP-10) for 24 h. After stimulation, the cells were collected for analysis of intracellular IFN-γ and TNF-α in CD4+ T, CD8+ T and NK cells. [file Image2.TIF]
