# Supplementary material for: Application of ImmunoScore Model for the Differentiation between Active Tuberculosis and Latent Tuberculosis Infection as Well as Monitoring Anti-tuberculosis Therapy
Source: Front Cell Infect Microbiol. 2017 Oct 30;7:457. doi: 10.3389/fcimb.2017.00457 (PMC5670161; doi:10.3389/fcimb.2017.00457)
Supplement: Supplementary file 3 [file Table1.DOCX]

**Supplementary Table 1.** Classification of the enrolled participants.

| **Categories** | **Definition** |
| --- | --- |
| HC | . Negative T-SPOT.TB result;  . No pulmonary symptoms or active disease. |
| LTBI | . Positive T-SPOT.TB result;  . No clinical or radiographic evidence of ATB. |
| ATB | . Positive T-SPOT.TB result;  (1) confirmed ATB: smear or culture or Mtb-specific PCR positive;  (2) probable ATB: Mtb not identified; Clinical findings (including histopathologic, cytological, or biochemical indexes) accordant with ATB and positive response to anti-TB treatment. |
| TR | . ATB treated with standard chemotherapy regimen for one to six months. |

HC, healthy controls; LTBI, latent tuberculosis infection; ATB, active tuberculosis; TR, patients undergoing anti-TB treatment.
